# An Oil Fraction Neural Sensor Developed Using Electrical capacitance Tomography Sensor Data

**DOI:** 10.3390/s130911385

**Published:** 2013-08-26

**Authors:** Khursiah Zainal-Mokhtar, Junita Mohamad-Saleh

**Affiliations:** School of Electrical and Electronic Engineering, Engineering Campus, Universiti Sains Malaysia, Nibong Tebal, Penang 14300, Malaysia; E-Mail: venconmigo1010@gmail.com

**Keywords:** neural network, electrical capacitance tomography sensor, two-component flow, generic system, multi-layer perceptron

## Abstract

This paper presents novel research on the development of a generic intelligent oil fraction sensor based on Electrical capacitance Tomography (ECT) data. An artificial Neural Network (ANN) has been employed as the intelligent system to sense and estimate oil fractions from the cross-sections of two-component flows comprising oil and gas in a pipeline. Previous works only focused on estimating the oil fraction in the pipeline based on fixed ECT sensor parameters. With fixed ECT design sensors, an oil fraction neural sensor can be trained to deal with ECT data based on the particular sensor parameters, hence the neural sensor is not generic. This work focuses on development of a generic neural oil fraction sensor based on training a Multi-Layer Perceptron (MLP) ANN with various ECT sensor parameters. On average, the proposed oil fraction neural sensor has shown to be able to give a mean absolute error of 3.05% for various ECT sensor sizes.

## 1. Introduction

In the oil industry, it is vital to measure fluids in oil pipelines for optimizing exploitation, production and transportation (*i.e.*, loading and unloading of oil tankers) where flow rate measurements are required. In a conventional component fraction measurement method, the complex multiphase flow mixture consisting of oil and gas, for instance, is separated into individual components. Then, Single-Phase Flow (SPF) meters (*i.e.*, orifice plate ones for gas and turbine meters for oil), are used to measure each single-phase flow separately [1,2]. This conventional approach requires the lucid use of separators. It has obvious limitations such as the use of bulky devices with high installation and maintenance costs. In addition, this process is intrusive and invasive. Due to these problems, devising a simple and direct method which is capable of measuring each component fraction directly without going through a separation stage is extremely attractive. One such method is the Electrical capacitance Tomography (ECT) technique which has become increasingly popular in process engineering applications, particularly for petroleum extraction and processing [1,3,4], advanced materials research [5,6], the food industry [7,8] and various chemical reactors [9,10].

The first ECT sensor system was developed in 1970s by the US Department of Energy in Morgantown, WV to visualize the solid-particle distribution in fluidized beds [11]. The first real-time industrial ECT system was developed at the University of Manchester Institute of Science and Technology (UMIST) in 1991 to measure multi-component flows such as gas-oil and oil-water in pipelines [1,11]. Since then, realization of the importance of ECT has extended research in the area to many other countries and various institutions worldwide. Research projects encompassing the ECT technique which include enhancement of the hardware systems [12–18], improvement of its image reconstruction techniques [19–26] and exploration for industrial applications [27–30], have evolved rapidly since the 1990s with tremendous progress achieved in the past decades.

The first image reconstruction algorithm for ECT, known as the Linear Back Projection (LBP), was developed by Xie *et al.* in 1989 [31]. This algorithm is relatively simple and computationally fast in reconstructing images for ECT, but the images produced appeared to be distorted due to the soft-field effect. This effect is worse when the difference in the permittivity of the imaged materials is very high, such as for gas-water flows [19–21]. This has led to inaccurate oil fraction estimations. Since then, researchers have keenly developed various image reconstruction algorithms such as the Landweber [32–34] and Tikhonov [19,23,35] algorithms to produce better tomograms. Research works in image-based ECT measurement using an Artificial Neural Network (ANN) have also been carried out to improve the inaccuracy of the tomograms. The work by Noralahiyan *et al.* has shown that it is feasible to use the ANN technique to reconstruct more accurate images [23–26], however, the image reconstruction process is time consuming due to the requirement for intensive computations of hundreds of image pixels and hence, it is only suitable for offline data processing [27].

The current work focuses on utilizing a direct method. Using this method, a more accurate calculation of oil concentration is expected, along with shorter processing times because no pixels are involved, thus making it possible to cut costs and increase efficiency. Previous work by Xie *et al.* [36] employed a direct method for oil concentration estimation by calculating the average normalized capacitance measurements. Although the method is simple and fast (*i.e.*, did not require image reconstruction), it has been found to be flow regime dependent in that different calibration methods are required for different flow regimes.

ANN is renowned as a robust artificial learning tool for solving numerous ECT-based problems; with applications ranging from pattern recognition of multiphase flows in pipelines to direct process interpretation without recourse to image reconstruction. Previous works by Duggan and York [15] and William and York [16] have proven the feasibility of using ANN in determining component fractions in pipeline flows without going through the image reconstruction stage. While the earlier works have only focused on two-component flows, Mohamad Saleh *et al.* [37,38], have developed an intelligent estimation system which estimates not only the fraction of two-component flows but also that of three-component flows.

Despite the success, all the ECT works have been based on fixed ECT sensor parameters, which in turn have produced non-generic neural oil fraction sensor systems. In order to build a generic neural oil fraction system, various ECT parameters can be considered. Several works [39–42] have been carried out in analyzing the sensitivity and resolution of ECT sensor on the basis of various primary electrode sizes. Analyses have found that primary sensors mounted as close as possible to expend the effective sensing space, possess high sensitivity but the resolution will be reduced. However, if the size of primary sensors is reduced, the resolution will increase but sensitivity decreases. These findings have demonstrated that primary electrode size plays an important role in determining ECT sensor's capability to produce better tomograms. Based on this phenomenon, this work focuses on training ANN using ECT data of various primary electrode sizes to produce a generic neural oil fraction sensor that is robust at estimating oil fraction based on a range of primary electrode sizes.

## 2. ECT System

Basically, an ECT system works based on Poisson's equation given by:

(1) ∇⋅(ε(x)∇Φ)=−ρ(x)

where *ε*(*x*) is the permittivity distribution, Φ is the electrical potential and ρ is the charge potential. Meanwhile, the electric field is given by:

(2) E=−∇Φ

By applying Gauss law, the induced charge at electrode *j* when electrode *i* is the source electrode can be calculated using:

(3) $$Q_{\text{ij}}=\oint_{\Gamma_{j}}\varepsilon \left(x\right)E\cdot \hat{n}\text{d}l$$

where Γ is the closed curve enclosing the detector electrode and *n̂* is the unit normal vector to Γ*_j_* Given charge *Q_ij_*, the capacitances for all possible pairs of electrode combinations can be calculated using the equation:

(4) $$C_{\text{ij}}=Q_{\text{ij}}/U_{\text{ij}}$$

where *U_ij_* is the voltage between the source electrode *i* and the detector electrode *j*.

The basic principle of ECT is to employ a desired number of adjacent rectangular electrodes (separated by a small gap) as sensors. The electrodes are made of a conductive material placed around some process equipment containing the materials to be visualised. Electrode capacitance sensors detect materials which are non-conductive or have different dielectric properties. If the two flowing materials have different permittivities, the electrical capacitance between all possible electrode pairs will change. The values of capacitance measurement depend on the dielectric constant value of each component in the mixture and their distribution inside the process equipment. The changes are measured. The next step is to obtain an image of such material distribution using the capacitance measurements with an appropriate image reconstruction algorithm.

**Figure 1.**
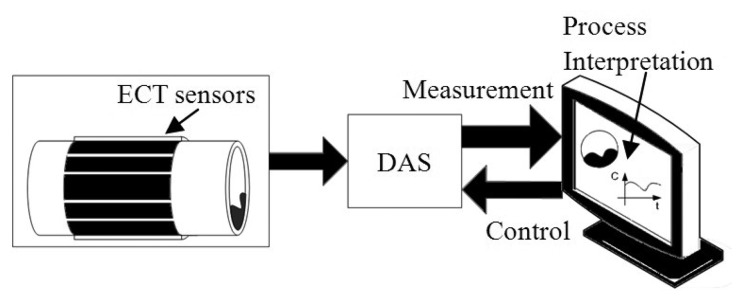
Schematic diagram of an ECT system.

The ECT system also consists of a data acquisition system (DAS) and computer system as illustrated in Figure 1. The DAS is responsible for measuring capacitance changes, converting the measured capacitance into a digital signal and sending the data to a computer system. The computer system is used to control the capacitance measuring operations and also produce the desired interpretation information, such as oil fraction and void fraction estimations.

Basically, an ECT system consists of a sensor, a data acquisition system (DAS) and a computer system consisting of various process interpretation components as illustrated in Figure 1. This work concerns the development of an intelligent interpretation component intended for oil fraction estimation. The following subsections discuss the sensor design and DAS utilized in this work.

### 2.1. ECT Sensor Design

The choice in the number of electrodes is a tradeoff between resolution, sensitivity and image capture rate. More sensing electrodes will produce better resolution images, but the measurement sensitivity will be lower because the size of electrodes becomes smaller and at the same time the capture rate is reduced. The sensitivity of the sensor and the image capture rate can be increased by using a bigger electrode size, though this will decrease the image resolution [43]. A study has found that 12 electrodes is a good compromise between sensitivity and resolution [44].

**Figure 2.**
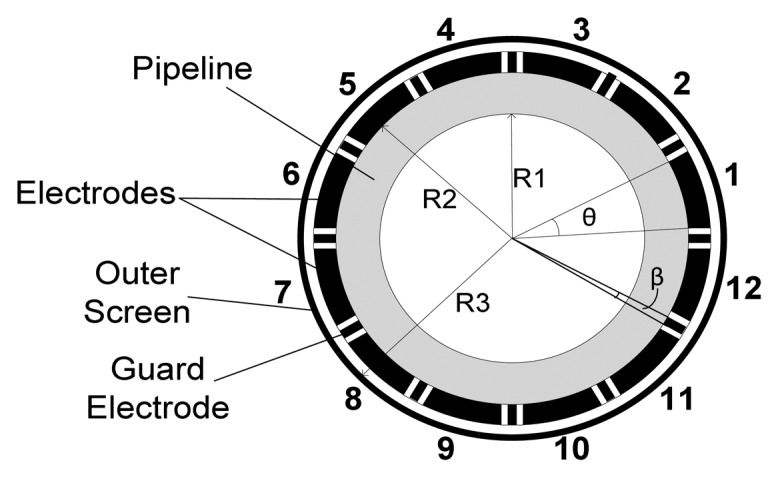
ECT sensor model.

Figure 2 shows a 12-electrode capacitance sensor. The inner pipe (R1), outer pipe (R2) and screen wall (R3) radii are 1, 1.06 and 1.30 units, respectively following previous research work on optimum pipe radii using the ECT simulator [41]. The guard electrodes (α) have a subtended angle of 2° while the primary electrode (θ) sizes is to be varied.

With 12-electrodes, there are 66 possible capacitance values that can be measured. This is based on:

(5) $$N=\frac{n\left(n-1\right)}{2}$$

where *N* is the total number of capacitance measurements and *n* is the number of electrodes.

As higher capacitance sensitivity is desirable and can be achieved by using larger angles of primary electrodes, the angles under investigation are chosen to be within the range of 20° until 26° with 0.5° interval. The minimum size of 20° is chosen because any smaller size of primary electrode leads to lower sensitivity distribution of the capacitance [35]. The largest electrode size of 26° is selected to allow sufficient space for 12 guard electrodes.

### 2.2. Data Acquisition

**Figure 3.**
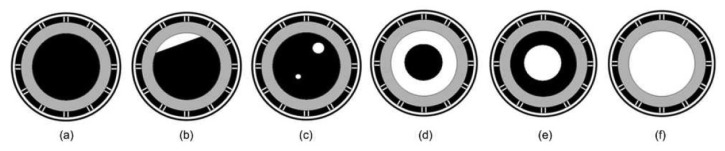
A schematic diagram of (**a**) full (**b**) stratified (**c**) bubble (**d**) core (**e**) annular and (**f**) empty flow regimes.

The ECT sensor data can be collected in two ways, by means of the actual plant data or generated from the ECT sensor simulator [45]. In this investigation, the ECT sensor simulator computes the changes in the capacitance measurements based on user-defined dimensions of the sensor parameters, relative permittivities of the materials within sensing area and geometrical distribution of the materials. There is no specific unit of sensor parameters used in this ECT simulator as it uses the coordinates unit for the dimension of the sensor parameters. In this way, the ECT sensor model can be easily changed to comply with desired application regardless of their length units. The correctness of thesimulated data obtained using the simulator has been verified in the work of Mohamad Saleh [46] based on real-plant ECT data. The simulated datasets are generated based on six flow regimes as depicted in Figure 3.

Various sets of ECT sensor data have been simulated based on different flow patterns of each flow regime. The numbers of simulated flow patterns for each flow regime are as depicted in Table 1. Empty pipe and full oil flows can only produce one pattern for each θ. These flows are used for the purpose of ECT sensor data normalization. Unlike the empty and full flows, stratified flow can be constructed from different oil heights as well as different tilt angles. Hence, many data can be generated based on stratified flow regimes. Similarly, bubble flow can be generated based on various bubble radii and different bubble locations, hence a large number of patterns can be produced based on the flow. Annular and core flows are simulated based on various central air and oil radii sizes. The fact is that these flows are just the opposite of each other, both flows have the same number of data.

This work proposes that each set of the measured capacitance is normalized based on the average capacitance measurement of empty and full flows of various θ ranging from 20° to 26°. This is because a generic oil fraction neural sensor system deals with ECT sensor data generated from primary electrode sizes, θ of 20° to 26°. Hence, every set of ECT sensor data corresponding to each flow pattern is normalized using:

(6) $$C_{\text{ij}}^{\text{norm}}=\frac{c_{\text{ij}}^{m}-c_{\text{ij}}^{\text{avge}}}{c_{\text{ij}}^{\text{avgf}}-c_{\text{ij}}^{\text{avge}}}$$

where $c_{\text{ij}}^{m}$ is the measured capacitance, $c_{\text{ij}}^{\text{avge}}$ is the average capacitance measurement of empty flow of various θ ranging within [20°, 26°] and $c_{\text{ij}}^{\text{avgf}}$ is the average capacitance measurement of full flow of various θ ranging within [20°, 26°]. This proposed normalization equation differs from the previous works of fixed θ, which uses:

(7) $$C_{\text{ij}}^{\text{norm}}=\frac{c_{\text{ij}}^{m}-c_{\text{ij}}^{\text{empty}}}{c_{\text{ij}}^{\text{full}}-c_{\text{ij}}^{\text{empty}}}$$

The sets of $c_{\text{ij}}^{\text{norm}}$ are used as inputs to an ANN. The corresponding outputs of the sets of $c_{\text{ij}}^{\text{norm}}$ are the oil fraction values. The term $c_{\text{ij}}^{\text{empty}}$ refers to the capacitance measurement of empty pipe of fixed primary electrode size θ and $c_{\text{ij}}^{\text{full}}$ is the capacitance measurement of full pipe of fixed-size θ.

The term component fraction, *f*_mat_ refers to the ratio of the cross-sectional area covered by the material of interest *A*_mat_, to the area of the entire cross-section of sensing area *A*_pipe_. In this work, the material to be estimated is oil and hence, the equation is given by:

(8) $$f_{\text{mat}}=\frac{A_{\text{mat}}}{A_{\text{pipe}}}$$

The values of component fractions range from zero (an empty pipe) to one (a pipe full either with oil or water). In this work, training and testing sets are chosen to be as large as possible and equal in numbers but of different sets. This approach has a potential to produce a more intelligent sensing system as larger number of training set is utilized and also to produce more accurate performance assessment as a larger number of testing datapoints is used [47,48]. Also, both datasets are made to have equal number of samples from each type of flow regime to create unbiased situation among the classes of flow regime. The generated ECT data are divided into training, validation and testing sets with a 4:2:4 ratio, respectively. To develop a generic neural estimator, the training, validation and test datasets corresponding to all the seven θs of ECT design are used. This is to ensure that the generic neural estimator can flexibly accept data obtained from all the seven primary electrode sizes. Then, each of the data in the three sets is normalized using Equations (6) and (7) to be used in the development of generic and non-generic neural oil fraction sensors, respectively.

## 3. Development of Neural Oil Fraction Sensor

### 3.1. Neural Sensor Architecture Setup

The robustness demonstrated by ANN in previous works has motivated the use in this work of a similar approach for developing a generic intelligent oil fraction sensor system. There are two types of learning with ANN; either supervised or supervised. The works by Mohamad Saleh *et al.* [37,38] used supervised learning for estimating component fraction, thus verifying that this type of learning is feasible for the estimation task. ANN supervised learning has three types of architecture: Single-Layer Feed-Forward (SLFF), Multi-Layer Feed-Forward (MLFF) and Recurrent. In this work, a variant of the supervised MLFF ANN known as the Multi-Layer Perceptron (MLP) is used due to its simple structure, but yet capable of solving variety of nonlinear problems including prediction task [49,50].

**Figure 4.**
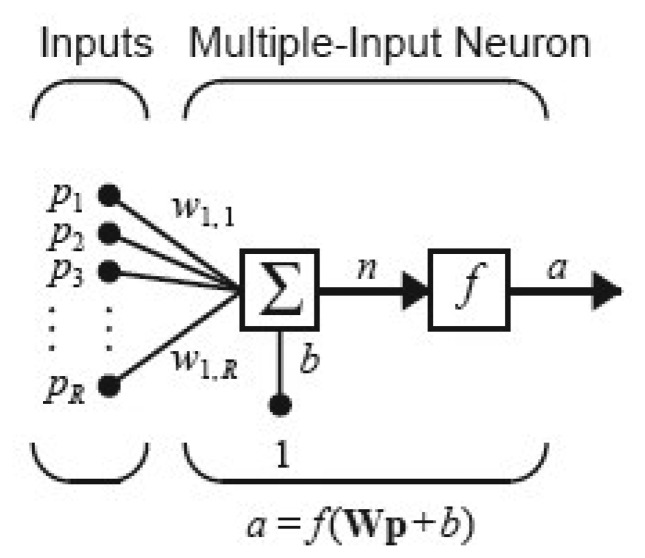
A multiple input neuron.

The MLFF ANN used in this work consists of multiple-input neurons as shown in Figure 4. Each individual input *p_1_*, *p_2_*, *p_3_*, …, *p_R_* is weighted by corresponding weights *w_1,1_*, *w_1,2_*, …, *w_1,R_*. The summation of weighted inputs and bias *b* form the net input *n*, can be described by following equation:

(9) $$n=w_{1,1}p_{1}+w_{1,2}p_{2}+\ldots +w_{1,R}p_{R}+b$$

In matrix form the equation can be written as:

(10) n=Wp+b

where for the single-input neuron case, the matrix **W** has only one row. Finally the output of a multiple-input neuron can be written as:

(11) a=f(Wp+b)

**Figure 5.**
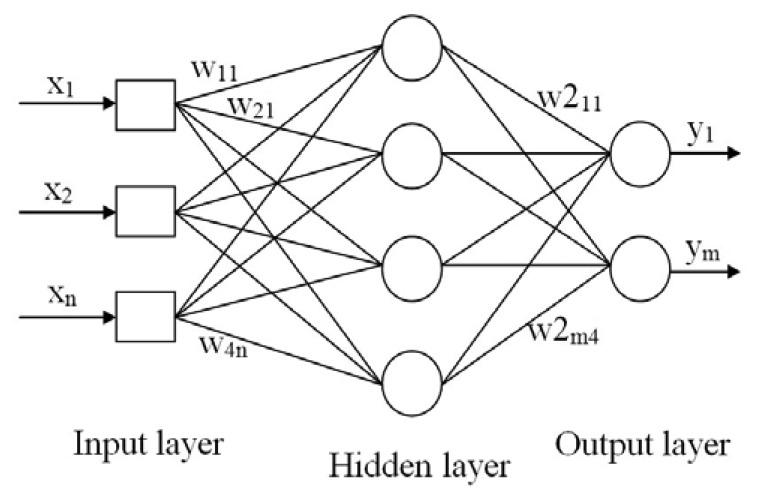
A schematic diagram of a Multi-Layer Perceptron (MLP) neural network.

These multiple-input neurons stack together to produce multiple layers that operate in parallel. Finally, these layers are cascaded together to form a fully-connected MLP network as depicted in Figure 5.

Figure 5 shows the architecture of a MLFF ANN. This ANN structure consists of three layers: the input, hidden and output layers, each containing a number of neurons, with vectors ***x***, ***w***, ***w2*** and ***y*** representing the ANN inputs, input weights, output weights and ANN outputs, respectively. Fundamentally, the input signals are fed into MLP through the input layer and these signals are passed to the hidden neurons via hidden weight connections. The hidden neurons perform some calculations and passed the computed output to the output layer through output weight connections. Further computations are carried out at the output neurons and final results are presented.

An MLP ANN with 66 input neurons (for 66 capacitance measurements) and one output neuron is constructed for developing a neural oil fraction sensor. The optimum number of hidden neurons is determined using a network-growing approach [51].

### 3.2. Neural Sensor Training Algorithm

The training process of an MLP is run based on a training algorithm. The Back Propagation algorithm [52] is a commonly employed training algorithm for MLP. There are several different Back Propagation algorithms with a variety of different computation and storage requirements. The selection of training algorithms is dependent upon the nature of the problem. For this work, two different training algorithms have been investigated: Levenberg-Marquardt (LM) and Bayesian Regulation (BR). These algorithms were chosen because of their success in the prediction and estimation fields [53–57]. The LM training algorithm is one of the most famous algorithms used for ANN learning in conjunction with back-error propagation error. It does not suffer from slow convergence rates which can lead to over-prolonged training times. This type of training algorithm combines the best features of Gauss-Newton algorithm and Steepest-decent method and avoids many of their limitations. This algorithm is very efficient when training small MLPs.

The BR training algorithm is an extended or modified version of the LM training algorithm. Although, this training algorithm updates weight and bias values according to LM optimization, it has a different performance cost function than LM training algorithm in that it also minimizes ANN error, along with weights as well as biases, to produce a network that generalizes well [58]. This algorithm has shown its capability to produce an ANN with smaller and smoother weights and biases so that over-fitting of training data can be prevented [59].

### 3.3. Neural Sensor Activation Function

A sigmoidal activation function is a nonlinear function that transforms the weighted sum of inputs to output values. It is applied to either hidden or output neurons. The choice of activation function for hidden neurons is important as the selection is usually problem-dependent and may affect an ANN's performance.

**Figure 6.**
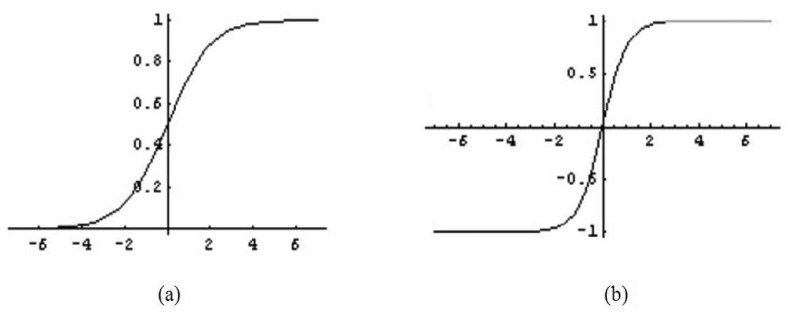
Activation function: (**a**) logarithmic sigmoid; and (**b**) hyperbolic tangent sigmoid.

As shown in Figure 6a, inputs for the logarithmic sigmoid activation function vary from −∞ to +∞ and are usually bounded by certain values (*i.e.*, the values of measurement data). This activation function squashes the inputs into the range [0, 1]. The expression of this activation function is given by:

(12) $$f\left(x\right)=\frac{1}{1+e^{-\text{kx}}}$$

where *x* is the sum of weighted inputs and *k* is the slope constant set to 1 in this work.

Another most common activation function used in Back Propagation learning is the hyperbolic tangent sigmoid activation function. This activation function is similar to the logarithmic sigmoid in that the input values range within [−∞, +∞], but it transforms into an output ranging from −1 to 1 as depicted in Figure 6b. The equation of the activation function is given by:

(13) $$f\left(x\right)=\frac{1-e^{-\text{kx}}}{1+e^{-\text{kx}}}$$

In this work, the output values for oil fraction can range from 0.0001 to 0.9999; therefore, the most suitable activation function to be applied at output neuron is logarithmic sigmoid (Logsig). Meanwhile, hyperbolic tangent sigmoid (Tansig) and logarithmic sigmoid (Logsig) functions are investigated to select the most suitable activation function for hidden neurons.

### 3.4. Neural Sensor Training

**Figure 7.**
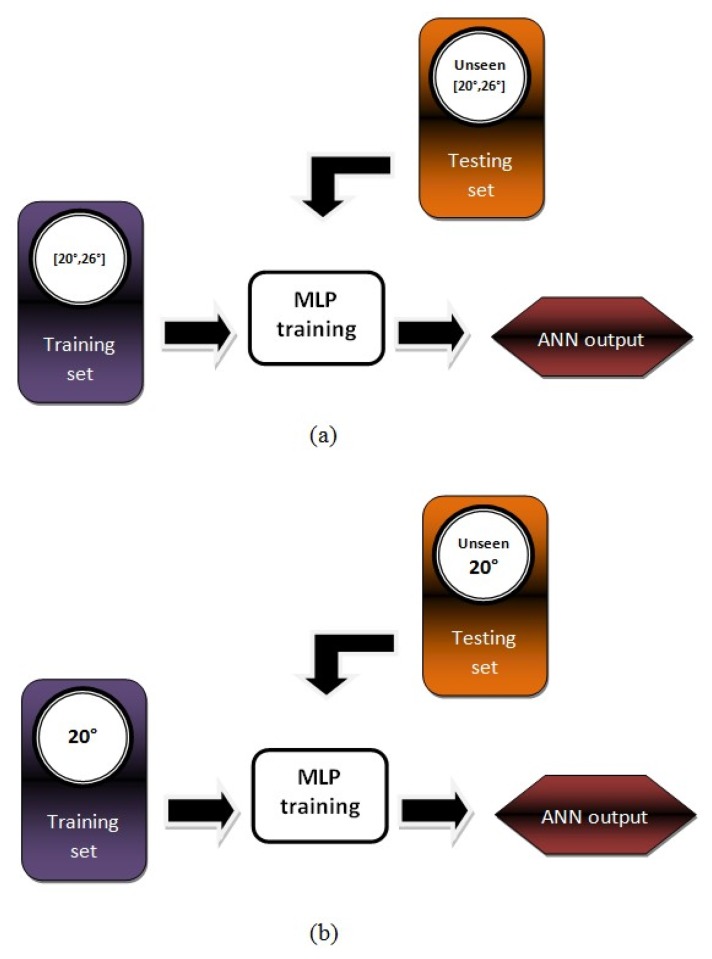
A schematic diagram of experimental method towards developing neural oil fraction sensors based on (**a**) generic θ; and (**b**) fixed-size θ.

An experimental method has been devised for our investigation towards developing a generic neural oil fraction sensor using various θ ranging from 20° to 26° with 1° intervals, as illustrated in Figure 7a. Also, for comparison purposes, seven non-generic neural oil fraction sensors are also developed and also to test the generic capability of the intelligent sensing systems for values of other θs. This method is also an ordinary method used by all previous works whereby the MLP is trained with ECT sensor data involving various flow patterns simulated with one θ and it is depicted in Figure 7b.

**Figure 8.**
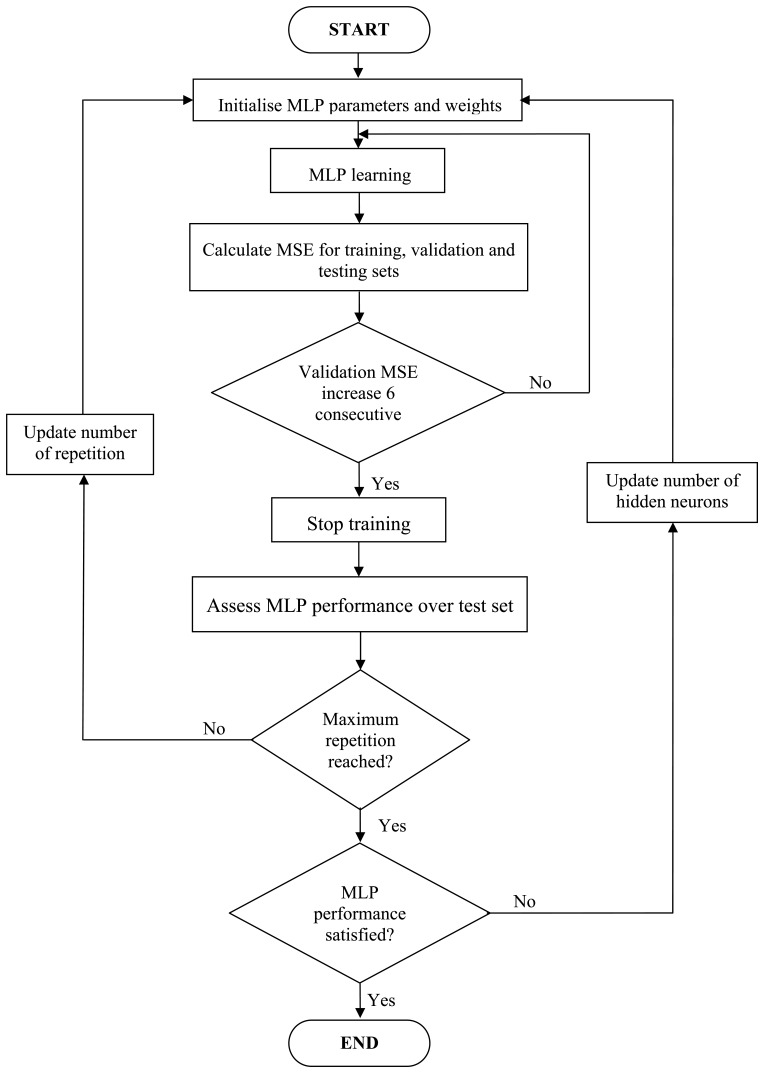
MLP training process to develop intelligent flow sensor.

Figure 8 shows the MLP learning process. The MLP ANN starts by initializing its weights with random values. Then, all the ECT electrode readings from the training set are presented to the input neurons. The MLP computed outputs are then recorded (*i.e.*, the characterization of the flow components) and compared with the actual or target outputs. The error difference between the MLP's and actual outputs are back-propagated to the earlier MLP layers to adjust the weight of links. The steps of presenting of training data and back-propagating of the errors are repeated until acceptably small errors have been reached or until the MLP has reached saturation (*i.e.*, no longer appears to be capably learning). At this point, the testing set is used to verify the trained MLP's ability to correctly predict the actual components fraction of each testing data. An estimation error is calculated as an indicator of the performance of the trained MLP.

#### 3.4.1. Development of a Generic Neural Sensor

To develop a generic neural network for oil fraction estimation, 2800 ECT data have been used and divided into 40% for training, 20% for validation and 40% for test sets. The raw data have been normalised using Equation (6). Besides normalised data, raw data have also been used to train the MLPs for comparison purpose. Two variants of the backpropagation algorithms; Levenberg-Marquardt (LM) and Bayesian Regularisation (BR) are put into trial for estimation task. Two sigmoidal activation functions namely the logarithmic sigmoid (Logsig) and hyperbolic tangent sigmoid (Tansig) functions are also utilised. Different MLPs are trained with combinations of the types of data, training algorithms and activation functions. Then, the performance of these MLPs are evaluated and compared.

The performance of a MLP oil fraction sensor is evaluated based on mean absolute error (MAE). This error measure is used because it gives good indicator of how much MLP estimations of oil fraction differ from their actual values. MAE is given by:

(14) $${\text{MLP}}^{\text{Best}}=\text{Min}\left(\frac{1}{P}\sum_{i=1}^{P}\left|T_{i}-O_{i}\right|\right)$$

where *P* is the total number of testing data, *T_i_* is the actual flow meter value for the *i^th^* testing data and *O_i_* is the MLP's estimation of flow parameter for the *i^th^* testing data. The best-performed MLP is selected based on the least MAE. The best training algorithm and the best hidden activation function for developing generic neural oil fraction sensor can also be determined from the results.

#### 3.4.2. Developing Non-Generic Neural Sensor

Non-generic neural sensors have been developed by separately training seven MLPs with ECT data with a single θ. A total of 160 training data, 80 validation data and 160 test data are used for each θ of the ECT design. The seven MLPs have also been trained using the best training algorithm and the best hidden activation function used to develop the generic neural sensor. Then,the evaluation of these non-generic neural sensors is carried out based on Equation (14). Eventually, the performances of the best-performing MLPs trained with generic and fixed-size ECT data are compared.

### 3.5. Enhancement of Neural Sensor Using PCA

**Figure 9.**
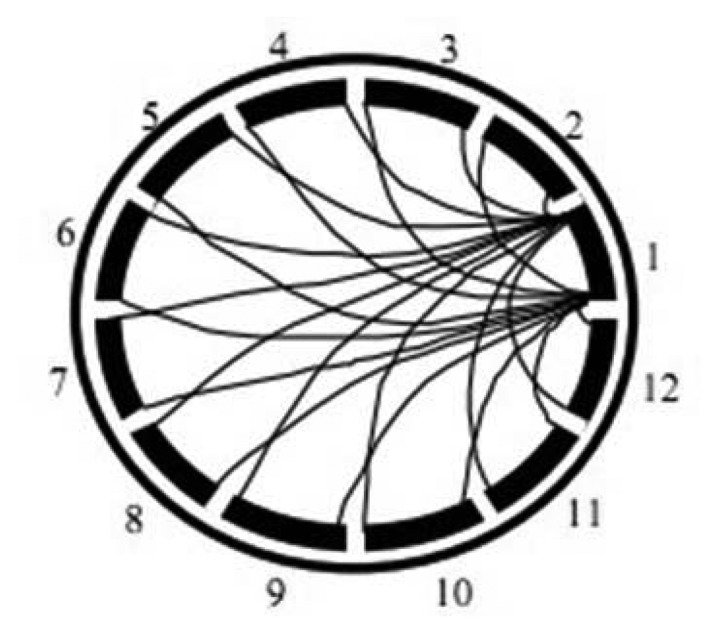
Overlapping sensing region between electrode 1 and other electrodes.

Upon determining the best procedure for developing a generic intelligent oil fraction sensor, the work proceeds with utilization of PCA technique on ECT sensor datasets. This step is an attempt to improve the capability of the generic neural oil fraction sensor. Basically, the PCA technique is applied to reduce correlated information in the ECT sensor data. This correlation in ECT sensor data creates confusion over MLPs during learning process, thus degrading their generalization capability. PCA is a procedure which converts a set of data of possibly correlated variables into a set of values of uncorrelated variables. The uncorrelated variables, for ECT is the capacitance measurement, are also known as the principal components. ECT sensor data have been known are highly correlated due to overlapping sensing areas as depicted in Figure 9.

**Figure 10.**
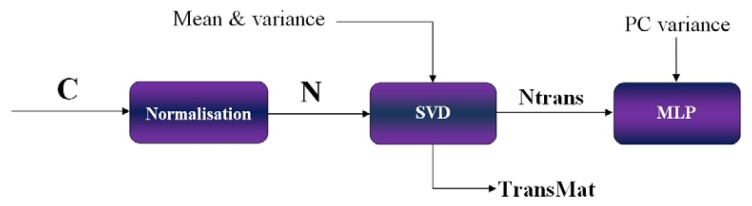
Schematic diagram of pre-processing of ECT data.

To implement the PCA technique for the ECT sensor data, raw ECT sensor data, C are first normalised to have zero mean and unity variance. Then using a mathematical technique called Singular Value Decomposition (SVD), along with the normalised ECT sensor data, N, mean and variance values the principal component are calculated. This generates a transformation matrix, **TransMat** and produces a transformed set of measurements which is the uncorrelated components, **Ntrans**. The transformation matrix, **TransMat** is stored for later use during the post-processing stage. The uncorrelated components, **Ntrans** are passed to the MLP with their corresponding target for the training process based on a chosen PC variance value. PC variance can be interpreted as a value of a fraction whereby the input components that contribute less than the fraction percentage of the total variation in the data set will be discarded. Several MLPs are trained with various PC variance values to find the optimum PC variance, *i.e.*, the one able to give the least MAE. The process explained above is known as the pre-processing stage and is depicted in Figure 10.

**Figure 11.**
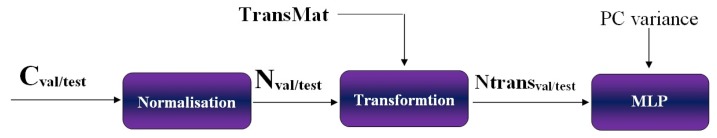
Schematic diagram of post-processing procedure.

While pre-processing is employed on the training set, validation and testing sets have to be post-processed using the post-PCA technique before they can be used for MLP training. Figure 11 illustrates the post-processing procedure. First, both data sets, **C_val/test_** are normalised. Next, the normalised data sets, **N_val/test_** are pre-processed along with the transformation matrix, **TransMat** that has been obtained during the pre-processing stage. This produces the new set of transformation matrices of uncorrelated data, **Ntrans_val/test_**. Then using PC variance values currently in use by the MLP undergoing the training process, a reduced set of uncorrelated ECT sensor data, **Ntrans_val/test_** are obtained. The trained-MLP uses this reduced uncorrelated data together with its optimum network weights obtain from the training process to estimate the oil fraction.

### 3.6. Verification of Generic Neural Sensor

The fully developed generic neural oil sensor needs to be assessed to verify its supremacy. The system is first evaluated based on test set which has been carried out during system development. The second evaluation involves executing the best-performed neural oil sensor using a verification dataset comprises 6,000 datapoints of various flows to further verify its performance. The verification data are ECT data obtained from θ values of 20.5°, 21.5°, 22.5°, 23.5°, 24.5° and 25.5°. These verification datasets are used to verify the robustness of generic neural oil sensor.

## 4. Results and Discussion

Table 2 shows the results for the MLP trained with raw and normalized ECT sensor data using different training algorithms and activation functions at the hidden layer. From the table it can be seen that MLPs trained using the LM training algorithm with a hidden neurons hyperbolic tangent sigmoid activation function are able to attain the smallest MAE values for raw and normalized data which are 4.13% and 3.73%. The results clearly indicate that the LM training algorithm is more appropriate for this work for it has shown its ability to jump out of the local minima trap in an error surface. As the choice of activation function is problem-dependent, the trial and error method in searching the best possible activation function for hidden layer has shown that hyperbolic tangent sigmoid activation function is the best choice. The experiments have also demonstrated that the suggested normalization approach can reduce the MAE from 4.13% to 3.73%.

Table 3 shows comparison results of intelligent oil fraction sensors trained based on fixed-size and generic θs. It can be seen that the performances of all MLPs trained with different fixed-size θs are not good. Their MAEs are larger than 6%. This is mainly due to their inability to generalize the test dataset which consists of ECT sensor data of different θs. The results suggest that MLPs trained with single-sized θ are not robust at solving interpretation based on ECT sensor data of other θ sizes.

**Figure 12.**
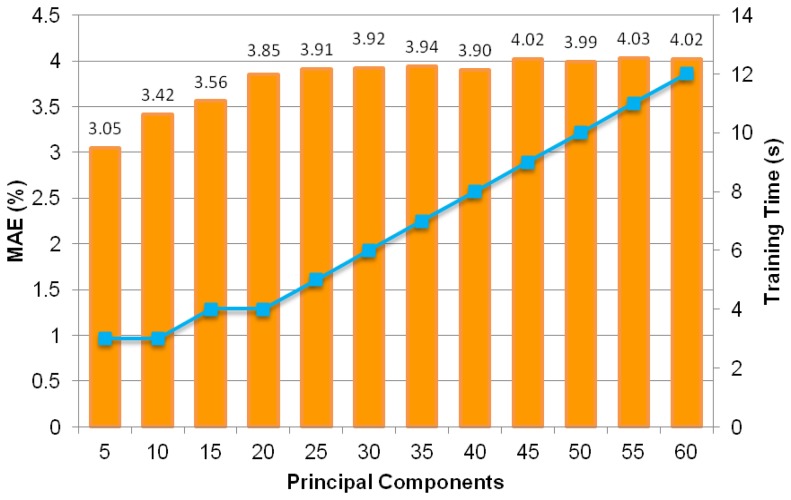
Test set MAE values and training times of MLP trained with five to 60 principal components.

Figure 12 shows MAEs of various MLPs that have been trained with uncorrelated data based on five to 60 principal components with five principal component intervals. It can be seen that the smallest MAE value at five principal components or input components is 3.05%. This MAE value is an improvement from without PCA after application of the PCA technique.

**Figure 13.**
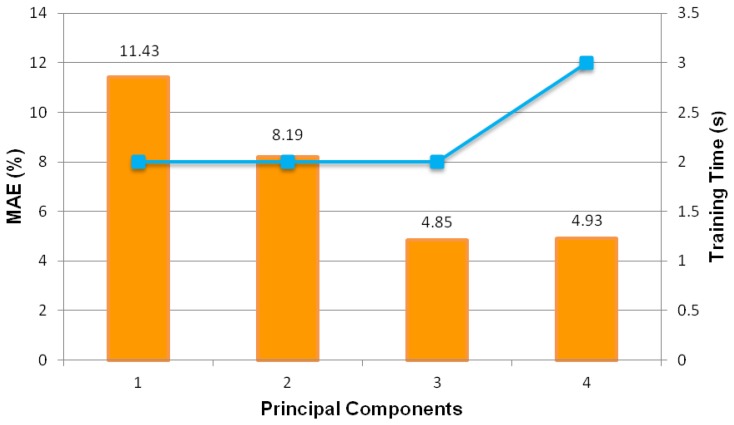
Test set MAE values and training times of MLP trained with one to four principal components.

**Figure 14.**
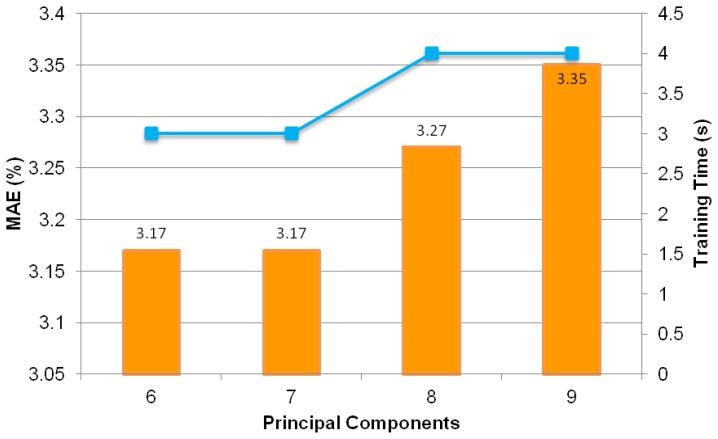
Test set MAE values and training times of MLP trained with six to nine principal components.

The search for the smallest possible MAE proceeds with MLPs trained with less than five input components (*i.e.*, from one to four principal components). As depicted in Figure 13, it can be observed that the smallest MAE of 4.85% is attained with four principal components. This MAE value is higher than the MAE of 3.05% obtained using five principal components. Further investigation is carried out to search for the optimum number of input components using six to nine principal components. The results are as shown in Figure 14, which clearly demonstartes that the lowest MAE of 3.17% is higher than the MAE of MLP trained using five principal components. Hence, five principal components is the optimum number of input components. It is able to produce the best-performed intelligent oil fraction sensor system.

Table 4 shows MAE of each flow regime and overall MAE of best-performed MLP before and after PCA technique is applied. The results clearly show that the MAE of the best-performing MLP reduces when the PCA technique is applied to the ECT sensor data.

Table 5 shows the results of MLP using optimal principal components to estimate the oil fraction from various flow regimes and different θs. The MAE values for 20.5°, 21.5°, 22.5°, 23.5°, 24.5° and 25.5° verification data are 1.65%, 1.63%, 1.70%, 1.99%, 3.03% and 4.15%, respectively. The results attained from the table are satisfactory as most MAEs obtained are below 3%. The results reveal that it is feasible to develop a generic neural oil sensor by employing MLP ANN.

**Figure 15.**
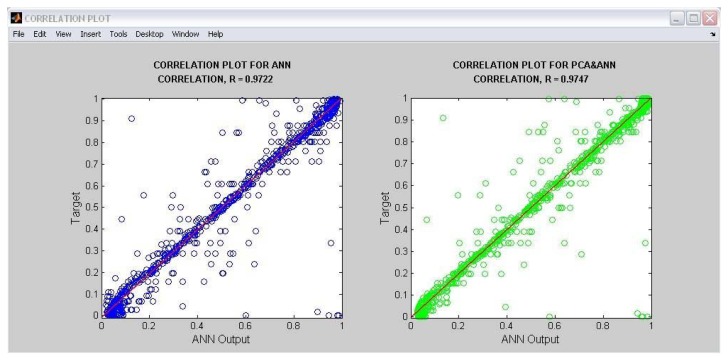
Correlation output display of oil fraction neural sensor supported by MATLAB.

Figure 15 shows the correlation plots, a function supported by code generation from MATLAB^®^. A correlation plot shows how close the MLP outputs are to the respective target outputs. From the figure, it can be seen that most data points gather closer to the y = x straight line going through the origin. This means that the MLP outputs have a high correlation with the target output values. The figure also shows that the MLP enhanced with the PCA technique has a higher correlation value, R of 0.9747, that the non-enhanced MLP with R = 0.9722.

## 5. Conclusions

Previous works in the literature only focused on developing a process parameter sensor based on fixed ECT sensor parameters. This paper has presented methods and experiments aimed at developing an intelligent oil fraction sensor system based on generic sensor parameters which is desirable in industry in order to save equipment and operation costs. The ANN technique is used to develop a generic neural oil fraction sensor using generic sensor parameters. The work proposed varying the primary electrode sizes towards developing the generic neural oil fraction sensor. It is also involved proposing a new generic normalization equation based on average empty and full ECT sensor data. Experimental methods have been devised for the investigation of the best developed neural sensor system. The results from the experiments have shown that it is feasible to develop a neural oil fraction sensor system for generic ECT data by employing MLP ANN. Furthermore, the application of PCA technique to the input data sets has shown its capability in improving the generalization performance of the MLP ANN. The MLP ANN generalizes better when the number of input datasets is optimum as the PCA technique has removed the correlated components. In the future, it would be interesting to consider other ANN architectures such as a Hybrid-MLP for the possibility of improving the performance of the neural oil fraction sensor system based on generic ECT sensor data. Besides that, the methods and experiments can be considered for the development of generic neural oil fraction sensor systems for three-component flows in a pipeline comprising gas, oil and water. This way the system could be made more useful as three-component flows are common in an oil industrial environment.

## Figures and Tables

**Table 1.** Number of flow patterns generated for each θ.

**Flow Regime**	**Number of Data**
Full	1
Stratified	1,224
Bubble	1,436
Annular	199
Core	199
Empty	1

**Table 2.** The MAE values for MLP oil fraction sensors trained using different training algorithms and hidden activation functions.

**Training Algorithm**	**Hidden Activation Function**	**MAE (%)**	

		**Raw Data**	**Normalised Data**
LM	Tansig	4.13	3.73
	Logsig	4.18	3.91

BR	Tansig	4.40	3.89
	Logsig	4.41	3.99

**Table 3.** The MAEs for different intelligent oil fraction sensors.

**Network**	**MAE (%)**
MLP_20°	7.56
MLP_21°	7.26
MLP_22°	7.84
MLP_23°	7.08
MLP_24°	8.46
MLP_25°	8.38
MLP_26°	6.30
Generic MLP	3.73

**Table 4.** MAEs of best-performed MLPs with and without PCA technique.

**Network**	**MLP**	**PCA & MLP**
MAE (%)	3.73	3.05

**Table 5.** MAEs of the best MLP using optimal principal components tested on ECT data based on various θs.

**Flow Regime**	**MAE (%)**					

	**20.5°**	**21.5°**	**22.5°**	**23.5°**	**24.5°**	**25.5°**
Stratified	2.63	2.62	3.02	3.74	5.34	8.16
Bubble	1.20	1.25	1.31	1.52	2.69	3.12
Core	0.99	1.15	0.95	1.07	1.63	1.52
Annular	1.50	1.19	1.03	0.96	1.48	2.26

Average	1.65	1.63	1.7	1.99	3.03	4.15
